# Antioxidants and Aquaculture: A Synergistic Approach for Sustainable Aquatic Production

**DOI:** 10.3390/antiox15070850

**Published:** 2026-07-05

**Authors:** Yukun Zhang, Amina Moss, Saichiro Yokoyama

**Affiliations:** 1 Faculty of Fisheries, Kagoshima University, Kagoshima 890-0056, Japan; 2 Institute of Aquaculture, University of Stirling, Stirling FK9 4LA, UK

## 1. Introduction

Global aquaculture is expanding to meet the increasing demand for high-quality aquatic protein, with projections indicating that the total production of aquatic animals will reach 205 million tonnes by 2032 [1]. However, the intensification of farming systems subjects aquatic organisms to multiple concurrent stressors, including high stocking densities, elevated ammonia and nitrite levels, temperature fluctuations, and opportunistic pathogens. These challenges are exacerbated by the escalating impacts of climate change, such as rising water temperatures, more frequent hypoxic events, and ocean acidification, which further threaten the health of aquatic animals and the stability of production. These diverse environmental and physiological stressors converge on a common cellular pathway: the excessive generation of reactive oxygen species (ROS) [2]. When ROS production surpasses the capacity of endogenous antioxidant defense mechanisms, oxidative stress occurs, resulting in lipid peroxidation, protein carbonylation, DNA damage, and tissue injury [3]. Chronic oxidative stress undermines immune competence, diminishes growth performance, impairs flesh quality, and increases mortality, thereby posing significant threats to the sustainability and profitability of aquaculture operations [4]. Climate change exacerbates these challenges by altering thermal regimes, intensifying hypoxic events, and promoting ocean acidification, particularly in coastal and brackish water systems where species such as the Pacific white shrimp (*Litopenaeus vannamei*) are cultivated [5,6].

In parallel with these biological challenges, the global regulatory landscape is increasingly moving away from the prophylactic use of antibiotics and synthetic chemotherapeutants in animal production, primarily due to concerns regarding antimicrobial resistance and environmental residues. The European Union’s Veterinary Medicinal Products Regulation (EU 2019/6), which came into effect on 28 January 2022, prohibits the prophylactic use of antimicrobials in groups of food-producing animals (Art. 107(1)) and has similar regulations in other jurisdictions [7]. China, the world’s largest aquaculture producer, banned the use of antibiotic growth promoters and related pharmaceutical feed additives in animal feed as of July 2020. Additionally, several Asian countries, including Thailand and Vietnam, have implemented national action plans on antimicrobial resistance that encompass aquaculture, and the Republic of Korea has imposed restrictions on their use of antibiotic growth promoters in animal feed as early as July 2011. These regulatory developments have created an urgent need for effective, environmentally compatible alternatives.

The global aquafeed additives market was valued at approximately USD 2.4 billion in 2025 and is anticipated to surpass USD 3.3 billion by 2033, with natural antioxidants and phytogenics emerging as the most rapidly expanding segment [8,9]. Functional feed additives, including vitamins, minerals, plant-derived phytochemicals, probiotics, postbiotics, and fermented agricultural by-products, have been identified as promising candidates to fulfill this role. Notably, several of these plant-derived materials have longstanding roots in traditional ethnopharmacological systems: isoflavone-rich soybean preparations are integral to East Asian fermented foods, longan peel (*Dimocarpus longan*) is utilized in Traditional Chinese Medicine for its “heat-clearing” and detoxification properties, and cannabidiol-rich hemp is employed in Southeast Asian folk veterinary medicine for enhancing stress resilience. This traditional knowledge provides an empirical foundation that complements the contemporary mechanistic framework discussed in this Special Issue. These natural antioxidants not only directly scavenge free radicals but also modulate evolutionarily conserved signaling pathways, such as the Nrf2-Keap1/ARE, AMPK, mTOR, and NF-κB pathways, thereby reinforcing mucosal barriers, optimizing metabolic pathways, and priming host immune defenses [10,11].

This Special Issue, “Antioxidants and Aquaculture: A Synergistic Approach for Sustainable Aquatic Production,” presents eight peer-reviewed original research articles that bridge molecular-level antioxidant mechanisms with practical aquafeed formulation. Collectively, these studies link molecular-level antioxidant mechanisms with growth, immunity, welfare, stress resilience, and post-harvest quality outcomes in commercially important finfish and crustacean species. Here, we provide an overview of the published articles and discuss their contributions to the field.

## 2. Overview of Published Articles

### 2.1. Dietary Phytochemicals and Micronutrients: Mitigating Metabolic Perturbations and Improving Muscle Quality

A central theme of this Special Issue is the exploration of dietary strategies to mitigate metabolic disturbances and enhance muscle quality. High-fat diets (HFDs) are increasingly utilized in commercial aquaculture due to their protein-sparing effect and improved feed conversion efficiency; however, they frequently lead to ectopic lipid accumulation, lipid peroxidation, and compromised flesh texture [12]. Xie et al. [Contribution 1] addressed this issue in the rice field eel (*Monopterus albus*), demonstrating that feeding an HFD (11.98% crude fat) resulted in significant lipid accumulation, disrupted myofibrillar arrangement, and deterioration of muscle quality. Dietary supplementation with 50 mg/kg soy isoflavones (SIFs) ameliorated these defects by activating the Nrf2 signaling pathway, up-regulating antioxidant enzyme genes (*sod*, *cat*, *gpx1*, and *gpx8*), and suppressing muscle lipid deposition. Concurrently, SIFs supplementation down-regulated myostatin (*mstn*) while up-regulating myogenic regulatory factors (*myod*, *tcap*, *mrf4*, and *mrf5*) and collagen synthesis genes (*ets1*, *sp1*, *p4ha1*). At 100 mg/kg, however, the beneficial effects were partially attenuated, indicating a dose-dependent response that echoes the hormetic pattern observed with cannabidiol supplementation in shrimp (see Section 2.3, Contribution 7). The study offers preliminary evidence to inform future high-fat aquafeed formulation with SIFs.

Water-soluble vitamins play an important role in maintaining immune homeostasis and alleviating stress responses in carnivorous species fed low-fishmeal formulations. Zhao et al. [Contribution 2] optimized low-fishmeal diets for juvenile largemouth bass (*Micropterus salmoides*) and evaluated their response to acute heat stress (33.0 ± 0.16 °C). Using quadratic regression analysis of specific growth rate (SGR), they determined the optimal L-ascorbic acid (vitamin C) supplementation level to be 0.77 g/kg. Adequate vitamin C levels reduced plasma transaminase (ALT and AST) leakage, enhanced systemic antioxidant capacity (T-AOC, GSH-Px, and CAT), and suppressed pro-inflammatory transcripts (*nf-κb*, *il-8*) while up-regulating anti-inflammatory *il-10*. Anti-apoptotic effects in gills and intestines, mediated through regulation of *bax*, *bcl-2*, and caspases, were confirmed by TUNEL fluorescence assays. These findings demonstrate that targeted vitamin C supplementation can enhance both growth performance and acute stress tolerance in fish fed low-fishmeal diets.

### 2.2. Probiotics, Postbiotics, and Fermented By-Products: Enhancing Mucosal Barriers and Gut Homeostasis

The interface between the intestinal microbiome, mucosal barriers, and systemic immunity represents an important target for functional feed additives. Wiratama et al. [Contribution 3] explored the synergistic application of the probiotic *Bacillus subtilis* AAHM-BS2360 and its postbiotic cell-free supernatant (CFS) in striped catfish (*Pangasianodon hypophthalmus*). The combined diet (Pro+Post) significantly enhanced growth parameters (SGR and ADG), elevated serum catalase activity, and boosted intestinal mucosal lysozyme activity. Gene expression analysis revealed systemic up-regulation of both immune-related transcripts (*lygl1*, *tgfb*, *b2ml*, *tnf*) and pro-inflammatory markers (*litaf*, *ifngr1l*, *c3*, *il13*, *il1b*). Following a challenge with *Edwardsiella tarda*, the Pro+Post group achieved the highest survival rate (87.5%), compared to 81.25% in the probiotic-only group and 62.5% in controls, demonstrating the immunomodulatory advantage of combined probiotic–postbiotic supplementation. The importance of mucosal barrier integrity observed here parallels the peritrophic membrane findings in organic acid-treated shrimp (see Section 2.3, Contribution 6), suggesting that reinforcement of epithelial barriers is a common mechanism across functionally diverse feed additives.

To overcome viability losses associated with probiotic processing and storage, Linh et al. [Contribution 4] provided the first report on alginate-encapsulated *Lysinibacillus* sp. PWR01—a spore-forming bacterium isolated from rubber latex nodules—in Nile tilapia (*Oreochromis niloticus*). Dietary inclusion of the encapsulated probiotic at 10^5^–10^6^ CFU/g over eight weeks improved final weight by 9.88% and reduced the feed conversion ratio (FCR) by up to 18.42%. The microencapsulated probiotic promoted intestinal development (increased villus height and width), enriched beneficial lactic acid bacteria, and selectively up-regulated pattern-recognition receptors (*tlr2*) and major histocompatibility complex class II (*MHC II*) without triggering chronic inflammatory cytokine cascades—a response pattern the authors describe as calibrated immuno-priming, characterized by selective PRR up-regulation in the absence of overt inflammation. This balanced immune activation resulted in 75.0% survival against the *Aeromonas hydrophila* challenge, compared to 45.0% in the control group.

The bio-valorization of agricultural by-products through fermentation represents a key strategy for supporting a circular bioeconomy in aquaculture. Wannavijit et al. [Contribution 5] evaluated the effects of fermented longan peel (FLP)—produced through enzymatic hydrolysis followed by *Lactiplantibacillus plantarum* TISTR 2265 fermentation—on Nile tilapia reared in a biofloc technology (BFT) system. Using quadratic modeling, the optimal FLP inclusion level was estimated at 20 g/kg. FLP supplementation enhanced in vitro carbohydrate and protein digestibility by increasing intestinal amylase, protease, and trypsin activities. It also improved intestinal histomorphology, stimulated mucosal and serum immune responses (lysozyme, peroxidase, and complement ACH50), and up-regulated growth-related (*igf-1*, *gh*, *npy-a*, *galanin*), immunoregulatory (*tlr-7*, *tnf-α*, *nf-κb*), and Nrf2-Keap1/ARE pathway (*hsp70*, *keap-1*, *nrf-2*, *gst-a*) genes. (KEAP1 serves as the negative regulator of NRF2; its co-regulation alongside *nrf-2* may reflect a compensatory feedback loop rather than a direct antioxidant effector mechanism.) This study highlights how biofloc systems can be integrated with functional feed additives to improve production efficiency.

### 2.3. Organic Acids, Phytocannabinoids, and Intrinsic Antioxidant Networks: Enhancing Stress Resilience and Product Quality

Aquatic invertebrates rely primarily on innate immune mechanisms, making them particularly responsive to targeted bioactive interventions. Pet et al. [Contribution 6] investigated the efficacy of a functional organic acid mixture (AuraAqua; Aq) against white spot syndrome virus (WSSV) in Pacific white shrimp (*Litopenaeus vannamei*). In vitro, Aq (2% *v*/*v*) reduced WSSV genome copies and decreased H_2_O_2_ release in primary gut cells without affecting catalase or superoxide dismutase expression. In vivo, dietary supplementation with 2% Aq reduced WSSV-induced mortality from 96.66% to 7.33%. This protective effect was mediated through multiple mechanisms: reinforcement of the peritrophic membrane (up-regulation of *mucin-1*, *mucin-2*, *mucin-5AC*, *mucin-5B*, and *mucin-19*), down-regulation of viral-entry pathways (lipopolysaccharide-β-1,3-glucan-binding protein, LGBP; and TLR signaling component LvECSIT), enhanced expression of cytoprotective heme oxygenase-1 (HO-1), and up-regulation of carboxypeptidase B (CPB) in gut and hepatopancreas tissue.

Peritrophic membrane integrity substantially enhanced Aq efficacy: infected shrimp with intact peritrophic membrane (PM+) had 9.34% mortality with Aq treatment, compared to 27.8% in peritrophic membrane-deficient (PM−) individuals. Given that WSSV infects shrimp via the oral route and must cross the peritrophic membrane to establish systemic infection, reinforcement of PM integrity through mucin up-regulation represents a mechanistically coherent antiviral defense. The up-regulation of HO-1—consistent with Nrf2-dependent regulation hypothesized in crustaceans—connects this prophylactic dietary antiviral approach to the Nrf2-mediated antioxidant mechanisms reported for soy isoflavones in rice field eel (see Section 2.1, Contribution 1), suggesting that Nrf2 axis activation may represent a conserved mode of protective action across vertebrates and invertebrates.

Phytogenic compounds can also act as adaptogens to enhance environmental stress resilience. Liu et al. [Contribution 7] evaluated dietary cannabidiol (CBD) in *L. vannamei* subjected to chronic ammonia nitrogen exposure followed by acute hypoxia. Their results confirmed a biphasic (hormetic) dose–response pattern. Moderate CBD inclusion (10–40 mg/kg) significantly improved growth and feed conversion ratio, enriched muscle polyunsaturated fatty acids (EPA and DHA), and restructured the gut microbiota by suppressing opportunistic pathogens. Under combined stress, moderate CBD prolonged survival by up-regulating hypoxia-inducible factor 1-alpha (*hif-1α*) and heat shock protein 70 (*hsp70*) while boosting systemic antioxidant capacity. Conversely, excessive CBD administration (80 mg/kg) induced hepatopancreatic toxicity, metabolic disruption, and compromised stress resilience, underscoring the importance of dosage optimization for phytogenic feed additives.

The relevance of intrinsic antioxidant networks extends beyond stress physiology to post-harvest product quality. Mittakos et al. [Contribution 8] compared muscle cellularity, fatty acid profiles, and oxidative stability of slow-growing (SG, <600 g) and fast-growing (FG, >1000 g) meagre (*Argyrosomus regius*) fillets during 10 days of cold storage at 2 °C. SG meagre exhibited significantly higher baseline muscle total antioxidant capacity (TOAC) and superoxide dismutase (SOD) activity compared to FG individuals. Despite the higher lipid content and elevated levels of docosahexaenoic acid (DHA) and total n-3 polyunsaturated fatty acids in SG fillets, no significant differences in malondialdehyde (MDA) accumulation were observed between SG and FG groups. This finding suggests that the elevated intrinsic antioxidant capacity in SG fish was sufficient to counteract the greater oxidative susceptibility of their lipid profile. In FG meagre, rapid hypertrophic muscle growth may have contributed to a relative depletion of antioxidant enzyme capacity at the cellular level, rendering fillets more vulnerable to postmortem lipid peroxidation.

### 2.4. Tissue-Integrated Metabolic Framework of the Eight Contributions

The eight studies span muscle (Contributions 1 and 8), gill and intestine (Contribution 2), gut (Contributions 3–5), hepatopancreas (Contributions 6 and 7), and systemic immune and antioxidant endpoints. These findings can be mapped onto a tissue-integrated metabolic framework in which the liver (hepatopancreas in crustaceans) serves as the central hub that integrates AMPK/mTOR/PPARα signaling and coordinates peripheral tissue responses.

In this framework: (i) HFD-induced myopathy in rice field eel (Contribution 1) is likely secondary to hepatic steatosis and impaired PPARα-mediated fatty acid oxidation, leading to systemic lipid overload; (ii) the heat-stress rescue by vitamin C in largemouth bass (Contribution 2) probably involves AMPK activation in the liver to maintain ATP homeostasis under thermal challenge; (iii) gut-derived signals from probiotics, postbiotics, and fermented products (Contributions 3–5) modulate hepatic metabolic programming through the gut–liver axis; (iv) peritrophic membrane reinforcement and HO-1 induction in shrimp (Contribution 6) reflect hepatopancreatic responses coordinated with gut barrier integrity; (v) CBD-mediated HIF-1α up-regulation (Contribution 7) induces glycolytic reprogramming that indirectly reduces mitochondrial ROS production—an antioxidant mechanism distinct from Nrf2 activation; and (vi) the growth–stress trade-off in meagre (Contribution 8) reflects mTORC1–AMPK antagonism, where high anabolic drive suppresses AMPK-dependent antioxidant transcription. This integrative perspective reveals that the Nrf2-Keap1/ARE pathway operates within a broader nutrient-sensing network, and that future research should target these integrative nodes with phosphoproteomic and metabolomic approaches.

**Figure 1:**
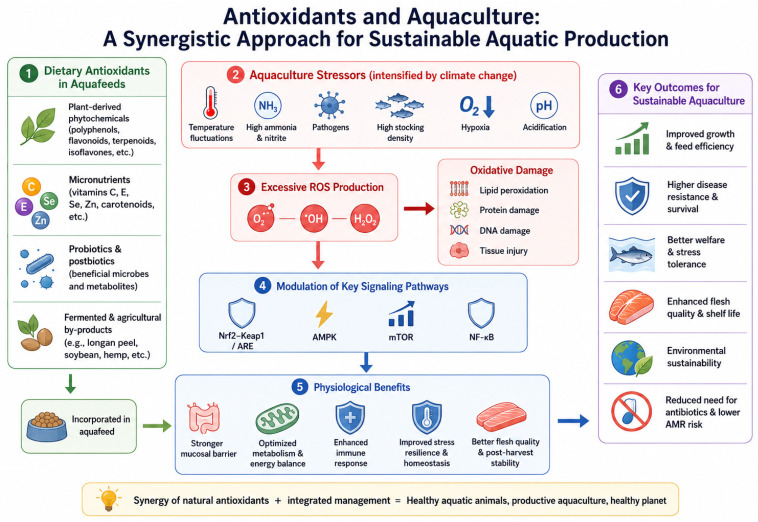
Summary of dietary antioxidant strategies for sustainable aquaculture. Functional feed additives (phytochemicals, micronutrients, probiotics/postbiotics, and fermented by-products) mitigate oxidative damage induced by aquaculture stressors via modulation of Nrf2-Keap1/ARE, AMPK, mTOR, and NF-κB signaling pathways, producing mucosal, metabolic, immune, and stress-resistance benefits that collectively support growth, health, welfare, product quality, environmental sustainability, and reduced antimicrobial reliance. Figure created with the assistance of ChatGPT 5.5 (OpenAI); the authors take full responsibility for the scientific content.

The conceptual framework described above is summarized in Figure 1, which integrates the four categories of dietary antioxidants, the aquaculture stressors that drive oxidative damage, the conserved signaling pathways that mediate protection, and the six sustainability outcomes supported by this Special Issue.

## 3. Conclusions

The articles assembled in this Special Issue collectively illustrate that modern aquaculture nutrition is advancing from empirical dietary supplementation toward targeted modulation of molecular homeostasis. Several cross-cutting themes emerge from these studies.

First, dose dependence is a recurring principle across diverse additive types—from soy isoflavones (50 mg/kg optimal, 100 mg/kg supra-optimal) and cannabidiol (10–40 mg/kg beneficial, 80 mg/kg toxic) to encapsulated probiotics (10^5^–10^6^ CFU/g optimal range) and fermented longan peel (20 g/kg optimal by quadratic modeling). This conserved hormetic pattern underscores the necessity of rigorous dose–response evaluation for any functional feed additive before commercial application, and highlights the value of quadratic regression approaches used across multiple contributions for establishing evidence-based inclusion levels.

Second, delivery technology is a critical determinant of efficacy. Microencapsulation protected spore-forming probiotics during processing and gastrointestinal transit, while the peritrophic membrane substantially enhanced the antiviral activity of organic acids in shrimp. The integration of functional additives within biofloc systems further emphasizes that the rearing environment and delivery matrix can amplify or attenuate the effects of dietary supplements. These findings highlight that formulation and delivery systems are as important as the bioactive compounds themselves. For heat-labile additives—particularly vitamin C, soy isoflavones, and other phytochemicals—post-pelleting bioactivity should be verified under commercial extrusion conditions (90–130 °C), and cold-pelleting (≤70 °C) or top-coating strategies should be considered to preserve efficacy where thermal degradation is confirmed.

Third, synergy occurs at multiple levels—both between co-occurring compounds within a single plant extract (intra-extract synergy) and between distinct additive types, as exemplified by the probiotic–postbiotic combination outperforming either alone. This points toward a future of multi-component functional feed formulations rather than single-additive strategies.

Fourth, intrinsic antioxidant capacity can be modulated by both genetic factors (growth rate, as shown in meagre) and nutritional interventions (vitamin C, soy isoflavones, CBD), with direct consequences for stress resilience, disease resistance, and product quality.

Fifth, the Nrf2-Keap1/ARE signaling pathway emerges as a convergent molecular theme across several contributions: soy isoflavones up-regulated *nrf2* transcription and downstream antioxidant enzymes in rice field eel; HO-1, a canonical NRF2 target gene, was induced by organic acids in shrimp; and multiple studies reported transcriptional regulation of Nrf2/Keap1 pathway components. This convergence suggests that Nrf2-Keap1/ARE agonism represents a promising mechanistic target for multi-species feed additive development, although definitive pathway activation evidence—such as nuclear NRF2 protein translocation or ARE-luciferase reporter assays—remains to be established in most of these models. The Nrf2-Keap1/ARE pathway does not operate in isolation; it is functionally integrated with central nutrient-sensing kinases—AMPK, mTORC1, and PPARα/γ—that directly connect nutritional status with oxidative metabolism, anabolism, and antioxidant defense. AMPK directly phosphorylates and stabilizes NRF2, inhibits mTORC1 to suppress anabolic demand during stress, and activates PGC-1α to drive PPARα-mediated fatty acid oxidation. Several findings in this collection—myogenesis and lipid metabolism in HFD-fed rice field eel, the hormetic dose–response patterns across multiple additive classes, and the growth–stress trade-off in fast-growing meagre—are consistent with AMPK–mTORC1–PPAR axis involvement. Future studies should investigate these pathways directly using phosphoproteomic approaches and target-specific inhibitors to disentangle the relative contributions of Nrf2-dependent and Nrf2-independent mechanisms.

Several important knowledge gaps remain. The cross-talk between Nrf2-Keap1/ARE and NF-κB pathways—and their integration with AMPK/mTOR nutrient-sensing networks—under multi-stressor scenarios requires further elucidation. The scalability of microencapsulation and fermentation-based by-product valorization technologies to commercial production levels needs validation through cost-benefit analysis, including formal Technology Readiness Level (TRL) assessment. Feed manufacturing stability—particularly the effects of extrusion temperature (90–130 °C) on heat-labile phytochemicals, the mechanical resilience of encapsulation matrices during pelleting, and the shelf-life stability of supplemented feeds under tropical storage conditions—remains inadequately characterized; post-processing bioactivity recovery should be reported as a standard validation step. The regulatory classification and approval pathways for these functional additives across major aquaculture markets—including the boundary between feed additives (Regulation (EC) 1831/2003) and veterinary medicinal products (Regulation (EU) 2019/6)—remain to be clarified and will influence commercial adoption, as will the intellectual property landscape surrounding proprietary strains and formulations.

For phytogenic additives specifically, the relationship between phytochemical composition, oral bioavailability in the target species, and in vivo efficacy is non-linear and requires dedicated pharmacokinetic studies; batch-to-batch standardization to marker compounds (e.g., HPLC-quantified isoflavone profiles, phenolic content) is essential for reproducibility. In particular, the oral bioavailability of polyphenolic compounds (genistein, daidzein, gallic acid, ellagic acid) after gastrointestinal transit in fish—including the requirement for intestinal deglycosylation of glucoside conjugates—remains poorly characterized and represents a critical gap between in vitro antioxidant potency and in vivo efficacy.

Statistically, the dose–response and quadratic regression results reported in several contributions should be interpreted with consideration of the limited number of tested dose levels (typically 3–4); larger dose-ranging studies incorporating confidence intervals and model diagnostics would strengthen the evidence base for the optimal inclusion levels identified.

From a welfare perspective, future studies should incorporate validated non-invasive welfare indicators (e.g., plasma cortisol, behavioral assessments) alongside molecular endpoints to confirm that functional feed additives genuinely improve the subjective welfare state of farmed aquatic animals.

The mechanistic leads identified here—particularly the conserved Nrf2-axis response across vertebrates and invertebrates and the hormetic dose-dependence patterns across multiple additive classes—provide a concrete molecular roadmap for the next generation of functional aquafeed design. We invite the broader research community to build on these findings toward effective, sustainable feed additives—including phytochemicals, probiotics, postbiotics, and fermented by-products—that support the global expansion of environmentally responsible aquaculture.

## Data Availability

The data presented in the individual contributions to this Special Issue are available in the respective articles or from the corresponding authors upon reasonable request. No new data were generated for this Editorial.
